# Evolving the HDRUK Phenotype Library: Phenotype Creation and Editing

**DOI:** 10.23889/ijpds.v9i5.2729

**Published:** 2024-09-18

**Authors:** Daniel S Thayer, Jack Scanlon, Muhammad A Elmessary, Artur Zinnorov, Ieuan Scanlon, Alex Coldea, Hannah Davies, Carla Oliveira, Spiros Denaxas, Emily Jefferson, Harry Hemingway

**Affiliations:** 1 SAIL Databank, Swansea University; 2 European Bioinformatics Institute; 3 Institute of Health Informatics, University College London; 4 Health Data Research UK

## Objective

The HDRUK Phenotype Library (phenotypes.healthdatagateway.org) shares definitions used to measure concepts of interest (such as diagnoses or treatments) within health datasets. It already holds more than 1000 phenotypes, with researchers able to contribute their work via an API. We aimed to create a more user-friendly method of contributing to the Library.

## Approach

We designed a phenotype creation workflow enabling users to create and submit new content via web interface. Goals included clarity and ease of use for a broad range of users.

## Results

A home page shows researchers’ own content. Researchers can create new phenotypes using a web form, entering metadata such as name, authors, description, and publications. Code lists are defined via one or more rules, including search terms or referring to another phenotype, or by CSV upload. Users can make their phenotypes accessible to a research group or to all authenticated users, as well as publish content on the web. Publication requests are reviewed to ensure content is complete and appropriate. Editing, with full history and version control, is also supported..

## Conclusions

We implemented and released the new features. We are currently engaging researchers to get feedback and invite content submission.

## Implications

The benefit of tools to support research transparency and repeatability is only realized when they are adopted. We hope that a GUI to support phenotype creation will broaden the Library’s user base help it serve as an enabler of higher-quality, more efficient research across the worldwide health data research community.

